# ImprimatinC1, a novel plant immune-priming compound, functions as a partial agonist of salicylic acid

**DOI:** 10.1038/srep00705

**Published:** 2012-10-04

**Authors:** Yoshiteru Noutoshi, Yusuke Jikumaru, Yuji Kamiya, Ken Shirasu

**Affiliations:** 1 Research Core for Interdisciplinary Sciences (RCIS), Okayama University, 3-1-1 Tsushima-naka, Kita-ku, Okayama, 700-8530, Japan; 2 RIKEN Plant Science Center, 1-7-22 Suehiro-cho, Tsurumi, Yokohama 230-0045, Japan

## Abstract

Plant activators are agrochemicals that protect crops from pathogens. They confer durable resistance to a broad range of diseases by activating intrinsic immune mechanisms in plants. To obtain leads regarding useful compounds, we have screened a chemical library using an established method that allows selective identification of immune-priming compounds. Here, we report the characterisation of one of the isolated chemicals, imprimatinC1, and its structural derivative imprimatinC2. ImprimatinC1 functions as a weak analogue of salicylic acid (SA) and activates the expression of defence-related genes. However, it lacks antagonistic activity toward jasmonic acid. Structure-activity relationship analysis suggests that imprimatinC1 and C2 can be metabolised to 4-chlorobenzoic acid and 3,4-chlorobenzoic acid, respectively, to function in *Arabidopsis*. We also found that imprimatinC1 and C2 and their potential functional metabolites acted as partial agonists of SA. Thus, imprimatinC compounds could be useful tools for dissecting SA-dependent signal transduction pathways.

Plants recognise pathogen-associated molecular patterns (PAMPs), such as flagellin and peptidoglycans, and induce PAMP-triggered immunity (PTI) to protect themselves from non-pathogenic microbes^1^. This defence mechanism is similar to the innate immune system in animals^2^. To suppress PTI and reproduce inside host plants, pathogenic microbes launch chemical or proteinaceous effector molecules. Plants perceive such pathogen-derived effector molecules via disease resistance (R) proteins, invoking effector-triggered immunity (ETI)^3^. ETI is a more rapid and stronger type of response than PTI, and it often results in the so-called hypersensitive response (HR), which consists of rapid induction of programmed death of infected cells that contain pathogens at the site of infection^1^.

Salicylic acid (SA) is a phytohormone that governs disease resistance responses in plants^4^. Exogenously applied SA can trigger defence responses, and it confers disease resistance against pathogens, including fungi, bacteria and viruses. Such enhanced disease resistance due to chemical inducers, referred to as plant activators, provides an effective method of crop protection^5^. Compared to commonly used pesticides that target pathogens, plant activators are not likely to be overcome by drug-resistant microbes because they affect host plants. Integration of plant activators into crop cultivation practices makes it possible to decrease both the volume and number of pesticides used. Thus, these compounds can provide a solution to avoid environmental contamination. Various types of compounds with stable and effective activities have been explored so far^6^. Benzothiadiazole (BTH) and 2,6-dichloroisonicotinic acid (INA) were developed as synthetic analogues of SA and BTH can be used in the field^7^,^8^. In contrast to such functional SA analogues, which directly induce defence responses, probenazole increases the potential for disease resistance responses in plants^9^. This immune-priming compound was discovered accidentally 35 years ago, and it is commercially available for the purpose of protecting paddy field rice from blast fungus and bacterial leaf blight^10^. The success of probenazole prompted the development of similar commercial drugs, such as tiadinil^11^ and isotianil^12^. These compounds have also been used as experimental tools to explore disease resistance mechanisms in plants. However, the precise modes of action of these molecules have not been identified.

The use of plant activators is still limited due to the phytotoxic effects when excessive doses are applied. These effects are likely caused by the strong induction of defence responses, which is associated with growth inhibition^13^,^14^,^15^,^16^. The limited disease spectrum of plant activators also restricts target crops. To obtain leads regarding compounds that could be used as plant activators, comprehensive surveys of chemical libraries have been conducted using various high-throughput screening methods^17^,^18^. We have established a high-throughput chemical screening procedure that allows selective identification of immune-priming agents, and we have obtained two important compounds, designated imprimatinA and imprimatinB^19^. These compounds cause immune priming by specifically perturbing the glucosylation of SA, which is a major metabolic pathway associated with defence responses^19^. In the present study, we characterised a novel immune-priming compound, imprimatinC1, which functions as a partial agonist of SA.

## Results

### Isolation of imprimatinC1 as a plant immune-priming compound

**Figure 1:**
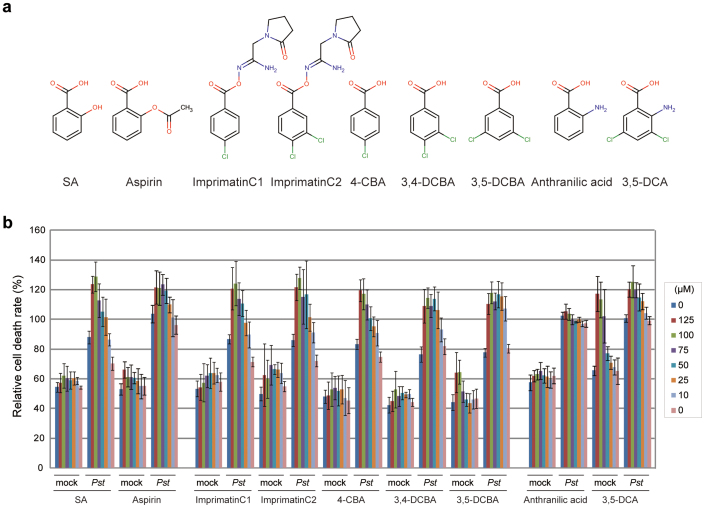
Cell death potentiation activities of the immune-priming compounds. (a) Molecular structures of the identified imprimatinC compounds and known plant immune-priming chemicals. (b) Bioactivity of the compounds regarding pathogen-induced cell death in *Arabidopsis* suspension cells. The compounds were incubated with *Arabidopsis* cells with or without the avirulent bacterial pathogen *Pst*-*avrRpm1* at the indicated concentrations, and the degree of cell death was measured quantitatively based on the concentration of Evans blue dye. Each cell death rate is shown as a value normalised to the mean of mock treatments with pathogens for each experimental group. Data are expressed as the mean ± SD (n = 4).

We surveyed a commercially available chemical library of 10,000 diverse small organic compounds using an established high-throughput screening method that allows selective identification of immune-priming compounds. Several chemicals, termed imprimatins, were successfully isolated. These compounds were found to potentiate cell death induced by the avirulent pathogenic bacterium *Pseudomonas syringae* pv. *tomato* DC3000 *avrRpm1* (*Pst-avrRpm1*) in *Arabidopsis* suspension cultures^19^. In this study, we characterised one of the isolated compounds, imprimatinC1 (Fig. 1a). As shown in Figure 1b, imprimatinC1 enhanced pathogen-induced cell death in *Arabidopsis* suspension cultures in a concentration-dependent manner, with no significant effects on cell viability being detected. The activity of imprimatinC1 was similar to the activities of SA and aspirin (Fig. 1a). We then performed a chemical structure search on the PubChem website using imprimatinC1 as a query and found a similar compound, which we designated imprimatinC2 (Fig. 1a). ImprimatinC2 also upregulated pathogen-induced cell death in a concentration-dependent manner (Fig. 1b). The cell death-potentiating activity of imprimatinC2 was slightly stronger than that of imprimatinC1 (Fig. 1b).

### ImprimatinC1 acts as a functional analogue of SA

**Figure 2:**
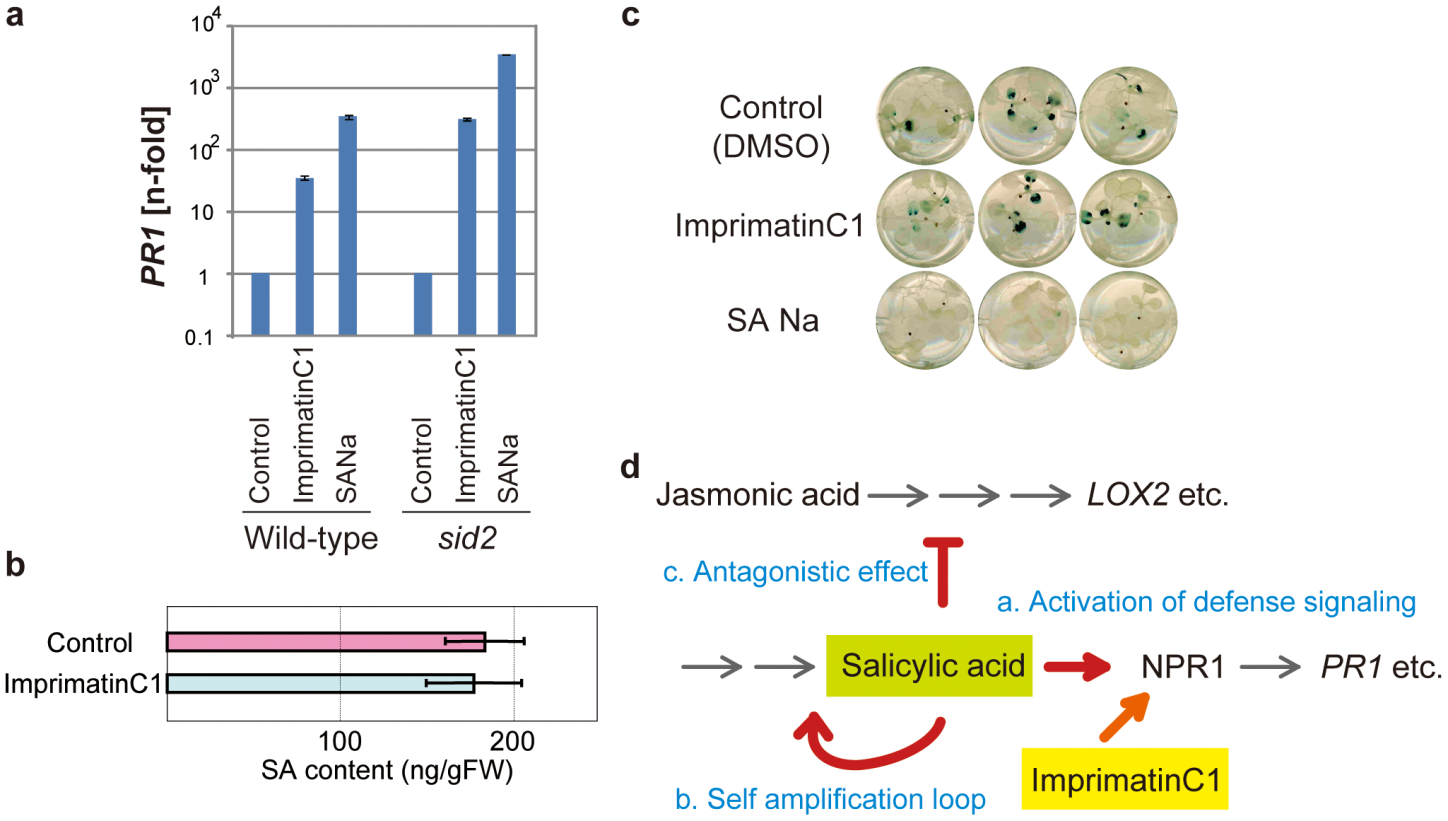
Activity of imprimatinC1 as an SA analogue. (a) Activity regarding defence gene expression. The *PR1* mRNA transcript levels were determined via qRT-PCR with cDNAs prepared from 10-day-old *Arabidopsis* seedlings (wild-type or *sid2*) soaked in liquid media containing 100 µM imprimatinC1 or SA for 24 hours. The expression values were normalised to *Actin2* as an internal standard. These results are representative of three independent replicates. (b) Activity regarding feedback regulation of SA synthesis. ImprimatinC1 (100 µM) was incubated with *Arabidopsis* cells in suspension for 24 hours, and the cellular SA content was measured via LC-MS. The data are expressed as the mean ± SD (n = 3). (c) Activity regarding JA signalling. A solution of 100 µM imprimatinC1 or SA was applied to transgenic *Arabidopsis* seedlings harbouring the *LOX2* promoter::*GUS* gene construct along with 100 µM MeJA for 24 hours, and GUS activity was determined. (d) Schematic representation of the pleiotropic effects of SA and the point in the pathway at which imprimatinC1 acts.

To examine the chemical properties of imprimatinC1, *Arabidopsis* seedlings were treated with 100 µM imprimatinC1 for 24 hours, and the transcription of *pathogenesis-related protein 1* (*PR1*), a marker gene for disease resistance responses, was analysed using real-time quantitative reverse transcriptase polymerase chain reaction (qRT-PCR). As shown in Figure 2a, imprimatinC1 induced *PR1* expression in a manner similar to SA, independent of pathogen challenge. ImprimatinC1 also induced the expression of *PR1* in *salicylic acid-induction deficient 2* (*sid2*) mutants (Fig. 2a). These results indicate that imprimatinC1 functions as an analogue of SA, but the ability of imprimatinC1 to induce downstream defence genes was weaker than that of SA.

### ImprimatinC1 only partially mimics the function of SA

SA signalling is known to be self-amplified^20^ (Fig. 2d). We addressed whether the endogenous content of SA is altered after the application of imprimatinC1 in *Arabidopsis* cells in suspension. As shown in Figure 2b, the cellular SA levels were not altered at 24 hours after chemical treatment, suggesting that imprimatinC1 is not likely to exhibit positive feedback activity related to SA synthesis. SA is also known to act negatively upon the signal transduction of the phytohormone jasmonic acid (JA)^21^ (Fig. 2d). To ascertain whether imprimatinC1 exhibits antagonistic activity toward JA, we monitored the expression of *lipoxygenase 2* (*LOX2*) after chemical treatment using *Arabidopsis* transgenic plants that contain the ß-glucuronidase (*GUS*) reporter gene driven by the *LOX2* promoter^22^. LOX2 is involved in JA biosynthesis, and its transcription is responsive to methyl jasmonate (MeJA)^23^. SA suppressed the induction of *LOX2* gene expression in response to MeJA treatment, whereas imprimatinC1 did not prevent the induction, even at a concentration of 200 µM (Fig. 2c). This result indicates that imprimatinC1 does not exhibit antagonistic activity against JA signalling. ImprimatinC1 can mimic only the downstream signalling properties of SA (Fig. 2d).

### ImprimatinC1 confers disease resistance to *Arabidopsis* plants

**Figure 3:**
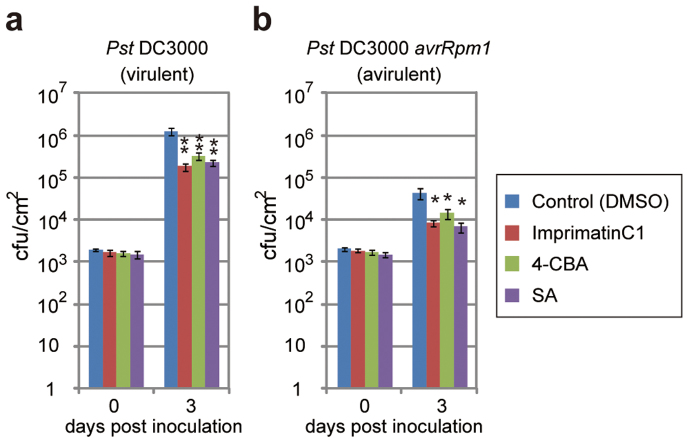
Disease resistance induced by imprimatinC1 and its potential active metabolite in *Arabidopsis* plants. *Arabidopsis* seedlings were grown for 4 weeks under short-day conditions and water containing 200 µM concentrations of the experimental compounds was sprayed onto the plants. As a positive control, 100 µM sodium salicylate (SA) was used. After 3 days, virulent *Pst* (a) and avirulent *Pst-avrRpm1* (b) were inoculated into leaves via syringe infiltration, and the numbers of bacteria were counted on the indicated days. Data are expressed as the mean ± SE (n = 4). (Student's *t*-test, **P < 0.01; *P < 0.05).

ImprimatinC1 was applied to *Arabidopsis* plants, and disease resistance was evaluated. Water with 200 µM imprimatinC1 was sprayed on *Arabidopsis* plants, and either virulent or avirulent strains of *Pst* bacteria were then inoculated into the leaves after three days. The bacterial counts in the leaves were determined on the indicated days post-inoculation. DMSO and 100 µM SA were used as negative and positive controls, respectively. Pretreatment with imprimatinC1 significantly decreased the growth of both bacterial strains (Fig. 3). These data indicate that imprimatinC1 functions *in planta* and increases disease resistance in *Arabidopsis* plants. The degrees of resistance conferred by 200 µM imprimatinC1 were similar to those obtained with 100 µM SA.

### The possible active moiety of imprimatinC1

**Figure 4:**
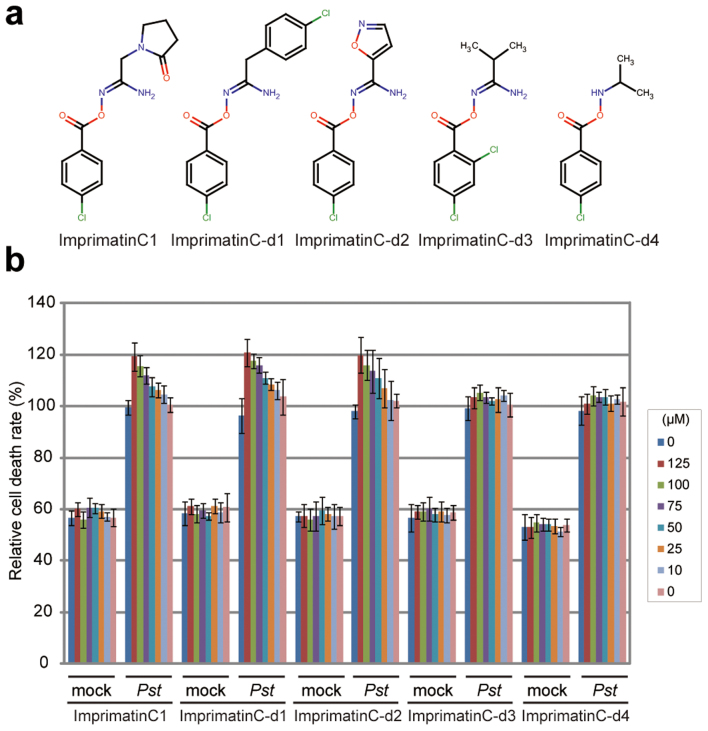
Structure-activity relationship analysis of imprimatinC1. (a) Molecular structures of the imprimatinC1 derivative compounds. (b) Bioactivities of the compounds regarding pathogen-induced cell death in *Arabidopsis* cells in suspension. Each cell death rate is shown as a value normalised to the mean value in mock treatments with the pathogen for each experimental group. Data are expressed as the mean ± SD (n = 4).

To determine which structural part of imprimatinC1 is important for its bioactivity, we explored structure-activity relationships using a series of molecules derived from imprimatinC1 (imprimatinC-d1 to –d5; Fig. 4a). ImprimatinC1-d1 and –d2 enhanced pathogen-induced cell death in suspension cells, similar to imprimatinC1 (Fig. 4b). These results suggest that the benzene ring shared by these molecules is responsible for their function. In contrast, imprimatinC1-d3 exhibited no activity, suggesting that a chlorine present at the 2-position of the benzene ring abolishes its activity (Figs 4a and 4b). ImprimatinC1-d4 also did not potentiate cell death, which means that the imine bond or the free amine group of this molecule is required for its activity (Figs. 4a and 4b). Thus, imprimatinC1 and its active derivatives possess an imine bond between their chlorinated benzene rings and their other side groups. It is known that particular imine structures that are associated with little steric hindrance can be hydrolysed with water^24^. Therefore, we determined whether the molecular structure of imprimatinC1 was altered in aqueous solution. The HPLC peak for imprimatinC1 dissolved in DMSO was completely identical to the peak obtained when the compound was solubilised in water for 24 hours, indicating that imprimatinC1 is stable in water (data not shown).

It was previously reported that the synthetic auxin sirtinol also contains an imine bond and is hydrolysed after incorporation into cells. One of its metabolites, 2-hydroxy-1-naphthaldehyde (HNA), can then be catabolised by a moco-containing aldehyde oxidase to become the active molecule 2-hydroxy-1-naphthoic acid (HNC)^25^. The results of our structure-activity relationship analysis suggest that an analogous transformation can occur in imprimatinC1 derivatives, and we hypothesised that imprimatinC1 may be broken into two smaller molecules and that 4-chlorobenzoic acid (4-CBA) might function as the active core of imprimatinC1. To test this possibility, we investigated whether 4-CBA can function as an immune potentiator, and we found that it promoted cell death in a way similar to imprimatinC1 (Fig. 1). Furthermore, 4-CBA conferred disease resistance against both avirulent and virulent bacteria in *Arabidopsis* to an extent similar to imprimatinC1 (Fig. 3). In this context, 3,4-dichlorobenzoic acid (3,4-DCBA) is thought to be the active subunit of imprimatinC2, and this molecule also potentiated pathogen-induced cell death (Fig. 1). It was found that 3,4-DCBA was more potent than 4-CBA, and this result is in agreement with the relationship between imprimatinC1 and C2 (Fig. 1). These results are consistent with our idea that imprimatinC1 and C2 can be metabolised *in vivo* to 4-CBA and 3,4-CBA, respectively, which are the active molecules.

Knoth *et al*., screened for chemical elicitors that induce the transcription of *At2g41090* (*CaBP22*), a gene belonging to the *Late/sustained Upregulation in Response to Hyalopernospora parasitica* (*LURP*) family, and they identified 3,5-dichloroanthranilic acid (3,5-DCA) as a positive hit in their screen^18^. In that study, derivatives of 3,5-DCA, including 3,5-dichlorobenzoic acid (3,5-DCBA), were also shown to induce the expression of *At2g41090* and confer disease resistance against *H. arabidopsis* Noco2^18^. The compound 4-CBA has been shown to cause weak induction of *At2g41090* gene transcription as well^18^. In our experiments, it was further demonstrated that 3,5-DCA and 3,5-DCBA enhanced pathogen-induced cell death in a concentration-dependent manner (Fig. 1). High concentrations of 3,5-DCA induced cell death, independent of pathogen inoculation. In contrast, 3,5-DCBA caused cell death potentiation, even at low concentrations, suggesting that it exhibits stronger activity than 4-CBA and 3,4-DCBA. Consistent with our results, 2,4-dichlorobenzoic acid (2,4-DCBA), a potential downstream metabolite of imprimatinC1-d3, presented no activity regarding the induction of *At2g41090* gene expression^18^.

### Induction of defence genes by imprimatinC1 and related compounds

**Figure 5:**
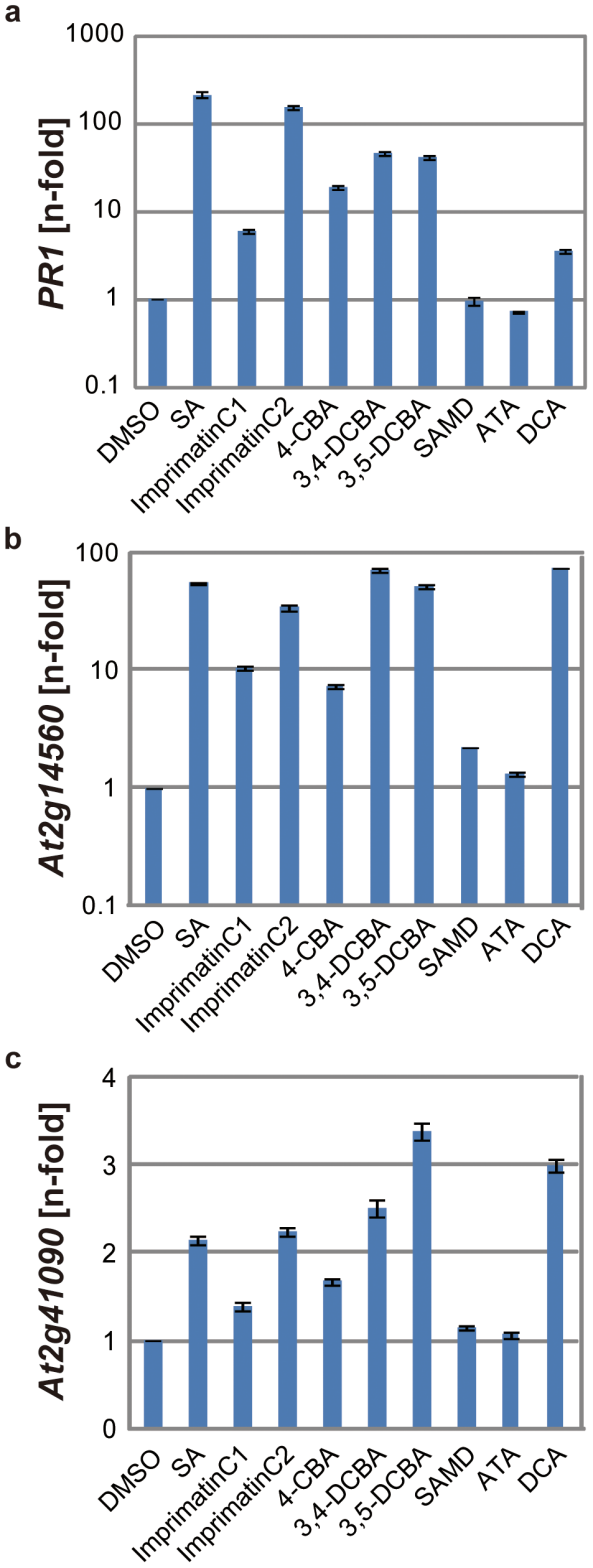
Induction of defence genes by imprimatinC1 and its derivative molecules. The mRNA transcript levels of *PR1* (a)*, At2g14560* (b) and *At2g41090*(*CaBP22*) (c) were determined by qRT-PCR with cDNAs prepared from 10-day-old *Arabidopsis* seedlings soaked in liquid media containing 100 µM concentrations of the experimental compounds for 24 hours. The expression values were normalised to *Actin2* as an internal standard. These results are representative of three independent replicates.

To characterise the downstream signalling pathway of imprimatinC1, we measured the expression of three SA-inducible defence-related genes via qRT-PCR in *Arabidopsis* seedlings treated with the appropriate chemicals for 24 hours. ImprimatinC1 induced transcription of the three genes, but the expression levels were lower than those obtained with SA (Fig. 5). The gene expression levels induced by 4-CBA were similar to those induced by imprimatinC1. ImprimatinC2 also induced expression of the three genes at levels similar to or lower than SA, but higher than imprimatinC1 (Fig. 5). The molecule 3,4-DCBA, the potentially active unit of imprimatinC2, behaved in a way similar to imprimatinC2 (Fig. 5). These results are consistent with the notion that imprimatinC compounds act via their downstream metabolites inside plant cells. It was observed that 3,5-DCBA also induced expression of three SA-responsive genes at levels similar to or greater than the levels obtained with 3,4-DCBA (Fig. 5). The compound 3-chlorobenzoic acid (3-CBA) was shown to exhibit stronger activity than 4-CBA regarding the induction of *At2g41090* gene expression 48 hours after application^18^. These results imply that the number and positions of chlorine atoms on the benzene ring of benzoic acid are critical for its bioactivity.

### ImprimatinC compounds function as partial agonists of salicylic acid

**Figure 6:**
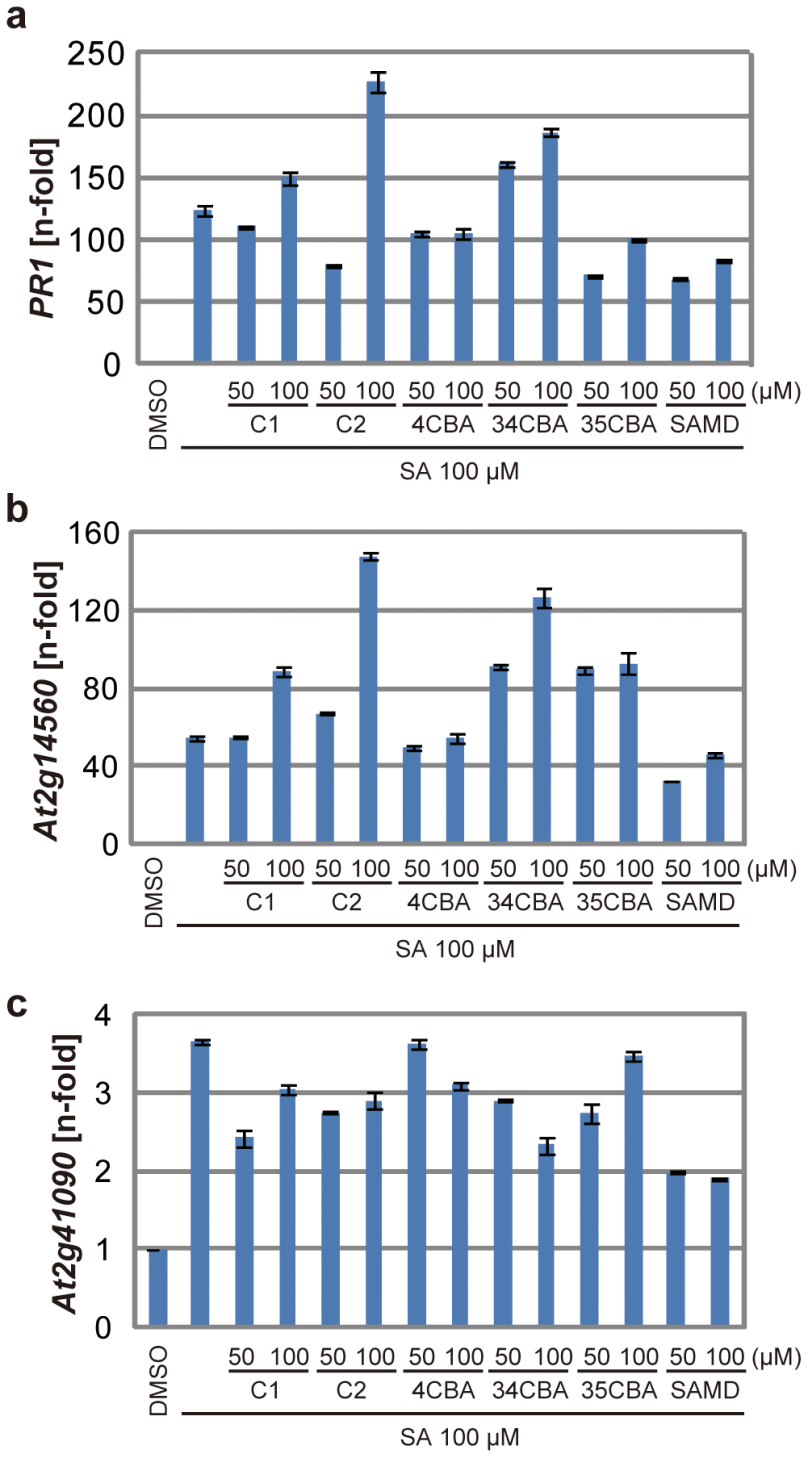
Activity of imprimatinC1 as a partial agonist of SA. The mRNA transcript levels of *PR1* (a)*, At2g14560* (b) *and At2g41090*(*CaBP22*) (c) were determined by qRT-PCR with cDNAs prepared from 10-day-old *Arabidopsis* seedlings soaked in liquid media containing 100 µM concentrations of the experimental compounds for 24 hours. The expression values were normalised to *Actin2* as an internal standard. These results are representative of three independent replicates.

The imprimatinC compounds and their presumed functional metabolites act as agonists of SA for downstream defence genes (Fig. 5). We addressed whether these compounds behave like partial agonists of SA. We applied 50 or 100 µM solutions of the compounds to *Arabidopsis* seedlings, in addition to 100 µM SA, and we analysed the expression levels of representative genes via qRT-PCR. As shown in Figure 6, these compounds did not exhibit simple additive effects with SA on the transcriptional induction of the three defence genes. The activities of these agonists for each gene appear to be prioritised over that of SA. This feature is a hallmark of partial agonists.

## Discussion

In this study, we isolated imprimatinC1 as a novel plant immune-priming compound from a commercial diversity-oriented library of 10,000 small molecules using a chemical screening system developed by our group^19^. ImprimatinC1 and its structural derivative imprimatinC2 promoted pathogen-induced cell death in *Arabidopsis* suspension cultures in a concentration-dependent manner, similar to SA, but these two compounds did not induce cell death without pathogen challenge (Fig. 1). Unlike imprimatinA and B, which target SA metabolism^19^, imprimatinC compounds exhibit weak SA-analogous activity and induce the expression of defence genes independent of pathogen challenge (Figs. 2a and 5). Because SA, itself, can function as a potentiator of HR cell death^26^, the identification of imprimatinC1 was entirely consistent with our experimental design. Here, we used a chemical library consisting of compounds with molecular weights ranging from 280 to 440. Thus, this library does not contain small derivatives of benzoic acid or SA, which could show SA-analogous activity, and this characteristic is what allowed us to isolate a novel SA analogue with a unique molecular structure. Interestingly, imprimatinC1 is unlikely to exhibit a positive-feedback effect on SA biosynthesis (Fig. 2b), and it did not compromise the MeJA-induced *LOX2* gene expression (Fig. 2c). This finding indicates that imprimatinC1 activity has only limited effects on downstream defence genes.

The results of the structure-activity relationship analysis supported our idea that imprimatinC1 and C2 can be converted to 4-CBA and 3,4-DCBA, respectively, most likely via metabolic conversion in *Arabidopsis* (Figs. 1 and 4). In fact, 4-CBA and 3,4-DCBA and their derivative 3,5-DCBA enhanced pathogen-induced cell death in suspension cultures (Fig. 1) and induced defence gene expression in *Arabidopsis* seedlings (Fig. 4). In both cases, imprimatinC2 and 3,4-DCBA exhibited stronger activities than imprimatinC1 and 4-CBA (Figs. 1 and 5), and the activity of 3,5-DCBA was stronger than that of 3,4-DCBA (Figs. 1 and 5). These results indicate that the activities of these benzoic acid derivatives as SA analogues depend on the number and position of chloride moieties in their benzene rings. Such structural differences could alter the affinities of these molecules for target proteins. Alternatively, chloride might affect the efficiency of the metabolism of these compounds. The electron-withdrawing activity derived from chloride may decrease the reactivity of carboxyl and/or hydroxyl groups on a benzene ring, which are targets of methylation^27^ or glycosylation^19^,^28^. Considering that imprimatinC1-d3 and its putative metabolite 2,4-DCBA did not show biological activity^18^, chloride moieties at the 3, 4 and 5 positions may be responsible for the binding of these benzoic acid derivatives to potential SA receptors.

We revealed that the identified imprimatinC compounds and their potential derivatives can function as partial agonists of SA and mimic the particular role of SA in downstream signalling (Fig. 6). Thus, imprimatinC compounds can serve as tools for the identification or validation of SA receptors. Partial agonists have previously contributed to the understanding of the precise modes of action of perception mechanisms and signal transduction mechanisms associated with small bioactive molecules, such as drugs and hormones. For example, the receptor of the phytohormone abscisic acid (ABA) was revealed using the partial agonist pyrabactin^29^. Because pyrabactin targets the specific molecules of multiple functional receptors, a chemical genetics approach can overcome the redundancy problem.

Most SA-inducible genes are controlled by the transcriptional activator NPR1^30^. NPR1 proteins are oligomerised by intramolecular disulphide bonding and are found in the cytosol. Upon SA treatment, NPR1 oligomers are monomerised due to a change in the intracellular redox status, and the NPR1 monomers are then translocated to the nucleus to activate gene expression^31^,^32^. Recently, NPR1 was shown to bind directly to SA through a metal (likely to be a copper) via two cysteine residues^33^. On the other hand, it was also shown that NPR3 and NPR4, which are paralogues of NPR1 but do not have the corresponding cysteine residues, bind directly to SA to control the SA-dependent degradation of NPR1^34^. It is possible that the affinities of imprimatinC compounds and their potential metabolites for NPR1/3/4 are different. Analysing the affinities of imprimatinC compounds for potential SA receptors could reveal the molecular basis of their insufficient and partial activities as SA agonists.

The commercially useful plant activators probenazole and tiadinil are metabolised in rice and converted to the bioactive substances BIT and SV-03, respectively^35^,^36^. Thus, these activators are prodrugs, which are useful because they are characterised by different absorption, distribution, metabolism and excretion properties in host crops compared with the original bioactive molecules. Stability is a desirable characteristic for agrochemicals, and applying a compound in the form of a prodrug can help extend its chemical efficacy. Accordingly, although we did not observe a clear difference in efficacy between imprimatinC and its potential metabolites in *Arabidopsis*, chemicals such as imprimatinC1 containing imine bonds might serve as tools for agrochemical development.

## Methods

### Chemicals

The following compounds were obtained from the indicated chemical vendors, as indicated: imprimatinC1 (Labotest (Bremen, Germany), LT00233283, [(E)-[1-amino-2-(2-oxopyrrolidin-1-yl)ethylidene]amino] 4-chlorobenzoate), imprimatinC2 (Labotest, LT01272595, [(E)-[1-amino-2-(2-oxopyrrolidin-1-yl)ethylidene]amino]3,4-dichlorobenzoate), imprimatinC1-d1 (ChemStar (Moscow, Russia), CHS1945076, (Z)-[1-amino-2-(4-chlorophenyl)ethylidene]amino 4-chlorobenzoate), imprimatinC1-d2 (MAYBRIDGE (Tintagel, Cornwall, UK), SPB03303, (Z)-[amino(1,2-oxazol-5-yl)methylidene]amino 4-chlorobenzoate), imprimatinC1-d3 (Vitas-M (Moscow, Russia), STK183095, [(1-amino-2-methylpropylidene)amino] 2,4-dichlorobenzoate), imprimatinC1-d4 (MAYBRIDGE, CD02160, cyclohexylidene amino 4-chlorobenzoate) and imprimatinC1-d5 (Vitas-M, STK094377, propan-2-yl amino 4-chlorobenzoate). The compounds 4-CBA, 3,4-DCBA, 3,5-DCBA and 3,5-DCA were purchased from Tokyo Chemical Industry (Tokyo, Japan). Anthranilic acid and aspirin were obtained from Wako Pure Chemical Industries (Osaka, Japan).

### Plant materials and growth conditions

*Arabidopsis thaliana* ecotype Columbia was used in this study. The transgenic *Arabidopsis* plants expressing *LOX2*promoter::*GUS* were described by Jensen et al.^22^. The plants were grown in MS agar medium or soil at 22°C under long-day conditions (16-h light/8-h dark cycles). To assay disease resistance, plants were grown in soil at 22°C under short-day conditions (8-h light/16-h dark cycles). *Arabidopsis* suspension-cultured cells were grown in a liquid medium containing MS with 3% sucrose, supplemented with 0.5 mg/L MES (pH 5.7), 0.5 mg/L naphthaleneacetic acid and 0.05 mg/L 6-benzylamino-purine under long-day conditions.

### Quantitative assay for pathogen-induced cell death

The methodology used in this assay has been described previously^19^. Data are represented as the mean ± SE (n = 4).

### Plant chemical treatments and RNA experiments

*Arabidopsis* seedlings (Col) grown in half-MS medium (1% sucrose) for two weeks were soaked in liquid half-MS medium (1% sucrose) supplemented with single or multiple chemicals at the indicated concentrations. The plants were incubated for 24 hours at 22°C, and the seedlings were collected in 2-ml tubes and frozen in liquid nitrogen. Then, samples were then crushed with four zirconium beads (*φ* 2 mm) using a homogenizer, Shake Master Neo (BMS, Tokyo, Japan). Total RNA was extracted using the PureLink Micro-to-Midi Total RNA Purification System, including an on-column DNase treatment step, as described in the manufacturer's protocol (Invitrogen, Carlsbad, CA, USA). To assess the RNA concentration and purity, a spectrometer (BioPhotometer plus; Eppendorf, Hamburg, Germany) was used. cDNAs were synthesised using the PrimeScript RT reagent Kit with gDNA Eraser (Perfect Real Time) (Takara, Shiga, Japan). The qRT-PCR amplifications were performed in 96-well plates using a LightCycler® 480 real-time PCR instrument (Roche Diagnostics, Basel, Switzerland) with the KAPA SYBR Fast qPCR Kit (KAPA BIOPUBLIC "-//NPG//DTD XML Article//EN"S, Woburn, MA, USA). Quantitation of the target transcripts was performed using the LightCycler 480 internal “Absolute Quantification 2^nd^ Derivative Max” software, and expression values were normalised with *Actin2*. The following primers were used in this study: 5′-CCGCTCTTTCTTTCCAAGC-3′ and 5′-CCGGTACCATTGTCACACAC-3′ for *Actin2*; 5′-TGATCCTCGTGGGAATTATGT-3′ and 5′-TGCATGATCACATCATTACTTCAT-3′ for *PR1*; 5′-AGAGTTTGCCAGGCTTATGATG-3′ and 5′-TCTCCAAAGTGCCACCAGTTG-3′ for *At2g41090*; and 5′-TCTTCACGGCAAGAGGATCT-3′ and 5′-ACCTGTCATGCATGGTCATC-3′ for *At2g14560*.

### Measurement of endogenous SA

Phytohormones were quantified using a 6410 Triple Quad LCMS (Agilent Technologies, Santa Clara, CA, USA) with an Agilent 1200 series rapid resolution liquid chromatography system fitted with a ZORBAX Eclipse XDB-C18 column (1.8 µm, 2.1 x 50 mm). d_6_-SA obtained from Sigma-Aldrich was used as an internal standard.

*Arabidopsis* cells in suspension treated with 100 µM imprimatinC1 or DMSO as a control for 24 hours were collected by centrifugation and frozen under liquid nitrogen. Then, samples were crushed with zirconium beads and extracted with 10 volumes of 80% (v/v) methanol containing 1% (v/v) acetic acid and the internal standard for 1 hour. The extracts were centrifuged at 14,000 x *g* at 4°C for 10 min, and the supernatant was collected. This procedure was repeated once, and the methanol was removed using a SpeedVac (Thermo Fisher Scientific, Waltham, MA, USA). Acidic water extracts were loaded onto an Oasis HLB extraction cartridge (30 mg, 1 ml; Waters, Milford, MA, USA) and washed with 1 ml of water containing 1% acetic acid to segregate highly polar impurities. SA was eluted with 2 ml of 80% methanol containing 1% acetic acid, and the methanol in this eluate was removed with a SpeedVac. Acidic water extracts were then loaded onto an Oasis MCX extraction cartridge (30 mg, 1 ml). After washing with 1 ml of water containing 1% acetic acid, SA was eluted with 1 ml of methanol containing 5% formic acid. After drying this fraction, 20 µL water was added, and the samples were analysed via LC-MS/MS.

### Chemical treatment and GUS staining

*Arabidopsis* seedlings harbouring the *LOX2*promoter::*GUS* construct were grown on MS agar plates for 10 days, and the plants were then immersed in liquid MS medium containing 200 µM imprimatinC1, sodium salicylate or DMSO in a 6-well plate for 3 hours. MeJA was then added to each well at a final concentration of 50 µM. After 5 hours of incubation, the plants were soaked in 90% acetone at −20°C for 1 hour. The samples were washed twice with 100 mM phosphate buffer (pH 7.4) and immersed in a staining solution containing 0.5 mg/ml X-gluc (5-bromo-4-chloro-3-indolyl glucuronide, in 100 mM phosphate buffer, pH 7.4, and 0.5 mM K^+^ ferricyanide/ferrocyanide) at 37°C. The staining buffer was then replaced with 70% ethanol for dehydration and depigmentation.

### Pathology test

The assay to determine disease resistance in the plants was performed as described previously^19^. *Arabidopsis* seedlings grown on MS agar plates for one week under short day conditions were transferred to rockwool and hydroponically cultivated at 22°C. After 3 weeks, the plants on the rockwool were transferred into small pots, and water supplemented with 100 µM concentrations of the experimental chemicals was added. The plants were then grown for 3 days prior to inoculation with pathogenic bacteria. Bacterial solutions containing *Pst* or *Pst-avrRpm1* were inoculated into leaves with a needleless syringe, and the number of bacteria in the leaves was quantified after 3 days.

## Author Contributions

Y.N. designed and performed the experiments and wrote the manuscript; Y.J. performed the SA measurements; Y.K. contributed to the experimental design, and K.S. designed the experiments and wrote the manuscript.
